# Accommodating version of a schematic eye for emmetropia and myopia

**DOI:** 10.1111/opo.13406

**Published:** 2024-10-25

**Authors:** David A. Atchison, W. Neil Charman

**Affiliations:** ^1^ Centre for Vision and Eye Research Queensland University of Technology Kelvin Grove Queensland Australia; ^2^ Division of Pharmacy and Optometry, Faculty of Biology, Medicine and Health University of Manchester Manchester UK

**Keywords:** aberrations, accommodation, myopia, peripheral refraction, raytracing, schematic eyes

## Abstract

**Aim:**

To develop an accommodating, wide‐angle, schematic eye for emmetropia and myopia in which spectacle refraction and accommodation level are input parameters.

**Method:**

The schematic eye is based on an earlier unaccommodated refraction‐dependent eye for myopia developed by Atchison in 2006. This has a parabolic gradient index lens and parameters derived from biometric and optical measurements on young adults. Several parameters are linearly dependent upon spectacle refraction (anterior radius of curvature of the cornea, axial length and vertex radii of curvature and conic asphericities of a biconic retina). The new accommodated schematic eye incorporates accommodation‐dependent changes in several lens‐related parameters. These changes are based on literature values for anterior chamber depth, lens thickness, vitreous chamber depth, lens surface radii of curvature and lens front surface asphericity. A parabolic variation of refractive index with relative distance from the lens centre is retained, with the same edge and centre refractive indices as the earlier model, but the distribution has been manipulated to maintain focus near the retina for the emmetropic case at 0 and 4 D accommodation. The asphericity of the lens back surface is changed so that spherical aberration and peripheral refraction approximately match typical literature trends. The model is used to compare spherical aberration and peripheral refraction in eyes with up to 4 D of myopia and 4 D of accommodation.

**Results:**

The levels of spherical aberration in the unaccommodated schematic eyes are similar to literature values for young adults, but the changes in spherical aberration with accommodation are approximately two‐thirds of that found in an experimental study. As intended, peripheral refractions in the accommodated schematic eyes are similar to those of their unaccommodated counterparts.

**Conclusion:**

The wide‐angle model extends the range of schematic eyes to include both refraction and accommodation as variable input parameters. It may be useful in predicting aspects of retinal image quality.

**Key points**

- An accommodating version of a wide‐angle, refraction‐dependent schematic eye has been developed, with changes in component lengths, lens shape and lens gradient index distribution.
- The accommodating schematic eye gives reasonable levels of spherical aberration and the intended peripheral refraction.
- The schematic eye has applicability in investigating peripheral image quality in spectacle lenses intended to slow myopic progression.

## INTRODUCTION

Following the early work of Gullstrand,^1^ several authors have developed models of the accommodating adult emmetropic eye.^2^, ^3^, ^4^, ^5^, ^6^ These models differ in their complexity, having different numbers of input parameters. The increase in prevalence of myopia has led to the development of a variety of spectacle and contact lens designs which attempt to control the incidence and progression of the condition. The effectiveness of several of these may be affected by ocular accommodation. This makes it desirable to develop an accommodating, wide‐angle, refraction‐dependent‐eye model so that the performance of myopia‐control methods can be better understood.

The refraction and accommodation‐dependent schematic eyes proposed in the present article are based on an earlier refraction‐dependent eye developed by Atchison,^7^ with parameters derived from biometric and optical measurements on young adults with refractions in the range of +0.75 to −12.38 D.^8^, ^9^, ^10^ This has a parabolic gradient index lens and six parameters that are linearly dependent on spectacle refraction, that is, the anterior radius of curvature of the cornea, axial length and four parameters of a biconic retina (vertex radii of curvature and conic asphericities in both *x* and *y* directions). The present study aimed to develop accommodated versions of the earlier unaccommodated schematic eyes, to cover from emmetropia to 4 D myopia and up to 4 D of accommodation. These eyes will be used for assessing multi‐segment lenses designed for the control of myopia. It was intended that the spherical aberration and peripheral refraction should be a reasonable match to those found in in vivo studies. The latter typically show that spherical aberration reduces with accommodation to become negative at about 2–3 D of accommodation,^11^, ^12^, ^13^, ^14^ and that accommodation has little effect on peripheral refraction.^15^, ^16^, ^17^, ^18^, ^19^, ^20^

## UNACCOMMODATED VERSION AND COMPARISON WITH LITERATURE

The parameters of the new refraction and accommodation‐dependent schematic eye are presented in Table 1. They are based mainly on results from several studies of ocular biometry as collated by Atchison and Smith.^21^

**TABLE 1:** General expressions for the parameters of the spectacle refraction (*SR*)‐ and accommodation (*A*)‐dependent schematic eye. Units for *SR* and *A* are dioptres. The expressions are valid for all combinations of *S* and *A* within the ranges 0 to −4 D and 0–4 D, respectively. Bolded parts of expressions indicate dependence on *SR* and/or *A*.

Medium	Refractive index	Vertex radius of curvature (mm)	Asphericity (*Q*)	Distance to next surface (mm)	Diameter (mm)
Air	1.0				
		7.77 **+ 0.022*SR***	−0.15		
Cornea	1.376			0.55	
		6.4	−0.275		
Aqueous	1.3374			3.15–**0.04*A***	
		11.48–**0.65*A***	−5 ‐ **0.4*A***		
Anterior lens	1.371 + *N*_0,1_ *Z* + *N*_0,2_Z^2^ + *N*_1,0_(*X* ^2^ + *Y* ^2^)^a^			*t*_1_ = 1.44 + **0.03*A***	*d* = 9.6–**0.259*A*** ^**0.81**^
		Infinity (i.e., surface is flat)			
Posterior lens	1.418 + *N*_0,2_Z^2^ + *N*_1,0_(*X* ^2^ + *Y* ^2^)^a^			*t*_2_ = 2.16 + **0.03*A***	*d* = 9.6–**0.259*A*** ^**0.81**^
		−5.9 + **0.17*A***	−2 ‐ **0.25*A***		
Vitreous	1.336			16.28 – **0.299*SR*** – **0.02*A***	
		*x*: −12.91 **– 0.094*SR*** *y*: −12.72 **+ 0.004*SR***	*x*: +0.27 **+ 0.026*SR*** *y*: +0.25 **+ 0.017*SR***		
Retina					

^a^ Coefficients *N*_*y*,*z*_ for the gradient index distribution are derived from the values of *t*_1_, *t*_2_ and *d*, using Equations (4a), (4b), (4c) and 5a‐5c (see text).

Atchison and Smith's Table 2.2 has anterior and posterior corneal vertex radii of curvature with unweighted means of 7.79 ± 0.12 and 6.36 ± 0.30 mm, respectively; the corresponding emmetropic schematic eye values of 7.77 and 6.40 mm are close to these numbers. Atchison and Smith's Table 2.3 has anterior and posterior corneal asphericities with unweighted means of −0.21 ± 0.10 and −0.45 ± 0.17, respectively; the corresponding schematic eye values of −0.15 and −0.275 are less prolate than the majority of studies. Doughty and Zaman's survey of the literature^22^ obtained a mean value of 0.536 ± 0.031 mm for the central corneal thickness; the schematic eye value of 0.55 mm is close to this.

Atchison and Smith's Table 2.4 includes lens parameters for the unaccommodated eye. The unweighted means of anterior and posterior radii of curvature are 11.78 ± 0.71 and 6.22 ± 0.36 mm, respectively; again, corresponding schematic eye values of 11.48 and 5.90 mm are close. The unweighted mean central thickness is 3.78 ± 0.11 mm, whilst the schematic eye value is slightly smaller at 3.60 mm. The unweighted mean lens diameter is 9.3 ± 0.4 mm, whilst the schematic eye value of 9.6 mm is near the limit of this range.

Lens parameters do not change with refraction in the schematic eye. This may differ slightly from the in vivo situation. Richdale et al.^23^ found the anterior surface radius of curvature to change at a rate of +0.11 mm/D of refraction (e.g., from 11.48 to 11.04 mm for 4 D of myopia), which means an increase in surface power with an increase in myopia. Like the change in the anterior corneal surface, the change in surface shape accentuates, although only to a small extent, the increase in axial length with the degree of myopia.

Overall, the unaccommodated schematic eye has most parameter values close to the middle of the range found in experimental studies. However, its corneal surface asphericities are underestimated, whilst its lens diameter is on the high side.

The gradient refractive index in the lens is given by the parabolic equation^4^

(1) $$n=1.418–0.037r^{2}$$

Here *r* is the relative distance, from the centre of the lens to its edge, of the point (*X*, *Y*, *Z*) lying within the lens, 1.418 is the refractive index in the centre of the lens and 1.418 − 0.037 = 1.371 is the refractive index at the edge of the lens. This equation can be changed to

(2) $$n_{a}=1.371+N_{0,1}Z+N_{0,2}Z^{2}+N_{1,0}\left(X^{2}+Y^{2}\right)$$

for the anterior half of the lens where the axial distance *Z* is measured relative to the anterior surface vertex and

(3) $$n_{p}=1.418+N_{0,1}Z+N_{0,2}Z^{2}+N_{1,0}\left(X^{2}+Y^{2}\right)$$

for the posterior part of the lens, where *Z* is measured relative to the lens centre. A flat ‘surface’ is introduced to separate the two gradient index distributions (the fifth surface in Table 1). The coefficients for the anterior part of the lens are

(4a) $$N_{0,1}=-2\times 0.037/t_{1}$$

(4b) $$N_{0,2}=-0.037/{t_{1}}^{2}$$

(4c) $$N_{1,0}=-0.037\times 4/d^{2}$$

where *t*_1_ is the central thickness of the anterior part of the lens and *d* is the lens diameter.

Corresponding coefficients for the posterior part of the lens are

(5a) $$N_{0,1}\;=\;0$$

(5b) $$N_{0,2}=-0.037/{t_{2}}^{2}$$

(5c) $$N_{1,0}=-0.037\times 4/d^{2}$$

where *t*_2_ is the central thickness of the posterior part of the lens.

## DEVELOPING AN ACCOMMODATED VERSION OF THE SCHEMATIC EYE

The nine factors to consider for change with accommodation are anterior chamber depth, anterior lens surface radius of curvature, anterior lens surface asphericity, lens central thickness, posterior lens surface radius of curvature, posterior lens surface asphericity, lens diameter, lens refractive index distribution and vitreous depth. To obtain the changes, literature values of change in parameter per dioptre of accommodation were considered.^23^, ^24^, ^25^, ^26^, ^27^, ^28^, ^29^, ^30^, ^31^, ^32^, ^33^, ^34^, ^35^, ^36^, ^37^, ^38^, ^39^, ^40^, ^41^, ^42^, ^43^, ^44^, ^45^, ^46^ Table 2 is a modification of Atchison and Smith's Table 2.5,^21^ with the addition of some recent studies^36^, ^37^, ^39^ and the inclusion of anterior chamber data. The data have been grouped according to whether accommodation demands (accommodation stimuli) or accommodation responses were specified. For the latter, in only one study was response measured concurrently with biometry^40^; other studies determined likely responses at similar times, usually by autorefraction. In most studies, the rates of change were determined by regression fits and hence are applicable only to the ranges of accommodation used, which in most cases extended to at least 4 D. Like the parameters for the unaccommodated eye, as far as possible, the results used are based on young adults, which is particularly important for studies using accommodation demands.

**TABLE 2:** Mean changes per dioptre in lens‐related parameters with either accommodation stimulus or accommodation response, from in vivo studies. Unit is mm/D, except for asphericity which uses D^−1^.

Stimulus/Response	Study	Anterior chamber depth	Anterior lens surface radius of curvature	Anterior lens surface asphericity	Central thickness	Posterior lens surface radius of curvature	Posterior lens surface asphericity	Lens diameter(mm)
Stimulus	Garner and Smith (1997)^24^	−0.029	−0.62		+0.035	−0.17		
	Strenk et al. (1999)^25^				+0.05			−0.08
	Koretz et al. (2002)^26^		−0.47					
	Dubbelman et al. (2005)^27^		−0.61	−0.5	+0.05	−0.13		
	Rosales et al. (2006)^28^		−0.6			−0.3		
	Tsorbatzoglou et al. (2007)^29^	−0.03			+0.04			
	Jones et al. (2007)^30^				+0.05			−0.07
	Kasthurirangan et al. (2011)^31^	−0.05			+0.05	−0.10		−0.07
	Ni et al. (2011)^32^	−0.02			+0.04			
	Doyle et al. (2013)^33^				+0.05			
	Pérez‐Merino et al. (2015)^34^	−0.04	−0.78		+0.04	−0.13		
	Khan et al. (2018)^35^				+0.07			−0.08
	*Martinez‐Albert* et al. *(2018)* ^36^	−0.04			+0.05			
	*Mitsukawa* et al. *(2020)* ^37^	−0.03	−0.51		+0.03	−0.05		
	Xiang et al. (2021)^38^	−0.03	−0.44		+0.03	−0.09		
	*Xie* et al. *(2022)* ^39^	−0.03			+0.04			
	**Mean ± standard deviation**	**−0.03 ± 0.01**	**−0.58 ± 0.11**	**−0.5**	**+0.05 ± 0.01**	**−0.14 ± 0.08**	‐	**−0.07 ± 0.01**
Response	Ostrin et al. (2006)^40^	−0.05			+0.07			
	Richdale et al. (2008)^41^				+0.05			
	Hermans et al. (2009)^42^		−0.6		+0.05	−0.15		−0.07
	Sheppard et al. (2011)^43^		−0.63		+0.08	−0.15		−0.09
	Richdale et al. (2013)^44^	−0.03			+0.06			−0.08
	Ramasubramanian and Glasser (2015)^45^	−0.05	−0.94		+0.07	−0.20		
	Richdale et al. (2016)^23^				+0.06			
	Martinez‐Enriquez et al. (2017)^46^	−0.04	−0.60	−0.36	+0.07	−0.22	+0.30	−0.14
	**Mean ± standard deviation**	**−0.04 ± 0.01**	**−0.68 ± 0.14**	**−0.36**	**+0.06 ± 0.01**	**−0.18 ± 0.04**	**+0.30**	**−0.09 ± 0.03**
	Navarro et al. (1985) schematic eye^3a^	−0.05/(*A* + 1)	−1.75/(*A* + 1)	−0.34/(*A* + 1)	0.1/(*A* + 1)	−0.23/(*A* + 1)	−0.125/(*A* + 1)	
	New schematic eye	−0.04	−0.65	−0.40	0.06	−0.17	−0.25	

*Note*: Unlike Table 1, both anterior and posterior lens surfaces have been assigned positive radii of curvature; steepening results in changes in the negative direction for both. Studies in italics do not appear in Atchison and Smith.^21^

^a^ The Navarro schematic eye also has an ‘equivalent’ lens refractive index with the rate of change of 0.00009 × (10 + 2*A*) D^−1^.A, accommodation.

Bolded values show means and standard deviations of the two groups of studies.

The rates of parameter change adopted to derive parameter values for the accommodated schematic eye are given in the bottom line of Table 2. They are biased towards the results of studies in which the accommodation response, rather than the accommodation demand, was specified. The lens of the unaccommodated schematic eye is divided into anterior and posterior parts with centre thicknesses 1.44 and 2.16 mm, respectively. Both of these increase by 0.03 mm/D (see Table 1). The anterior chamber depth decreases by 0.04 mm/D, the lens increases by 0.06 mm/D and the vitreous depth decreases by 0.02 mm/D.

Equation (1) is retained for the gradient index. Coefficients *N*_0,1_ and *N*_0,2_ change according to the increases in anterior and posterior part thicknesses *t*_1_ and *t*_2_. The diameter of the lens *d* has been altered so that the coefficient *N*_1,0_ in Equations (4c) and (5b) changes to keep the paraxial marginal ray height near zero for the emmetropic eye at a range of accommodation levels. The fit to the diameter *d* is

(6) $$d=9.6–0.259A^{0.81}$$

where *A* is the accommodation. The diameter varies from 9.6 mm for the unaccommodated eye to 8.804 mm for 4 D accommodation. It should be admitted that this is not particularly physiological as it is about twice most results given in the last column of Table 2.

Only two studies and one study, respectively, were available for anterior and posterior asphericities.^27^, ^46^ An asphericity change of −0.4/D was adopted for the anterior surface, but rather than using a ‘biometric’ posterior asphericity, we set the change for this as −0.25 mm/D to achieve the desired levels of spherical aberration and peripheral refraction (see the Results section).

## RAYTRACING AND ABERRATIONS

Analysis was performed with the OpticsStudio programme version 02.01 (Ansys Zemax, ansys.com/products/optics/ansys‐zemax‐opticsstudio). On‐axis spherical aberration and peripheral refraction out to a 45° field angle were determined for refractions between 0 D (emmetropia) and −4 D (4 D myopia) and accommodation values up to 4 D.

The Zernike spherical aberration coefficient was obtained by into‐the‐eye raytracing from an object point conjugate with the retina of the uncorrected eye. This approach was used because in practice aberrations are determined by out‐of‐the eye raytracing from the retina. The aperture size was manipulated to give a 5.0 mm entrance pupil for ease of comparison with experimental results.

Peripheral refractions were determined by tangential and sagittal raytracing out of the schematic eyes. In order to do this, the optical elements were reversed.

## ACCOMMODATION LEVELS

In this work, no distinction is made between accommodation stimulus and the corresponding ocular response. Of course, this is often not the case for real eyes, which may exhibit a lag or lead of accommodation with magnitudes depending upon the observing conditions. It can be dealt with as necessary in the modelling by having the stimulus set to one accommodation level and the schematic eye parameters set to another, usually lower, level.

**FIGURE 1:**
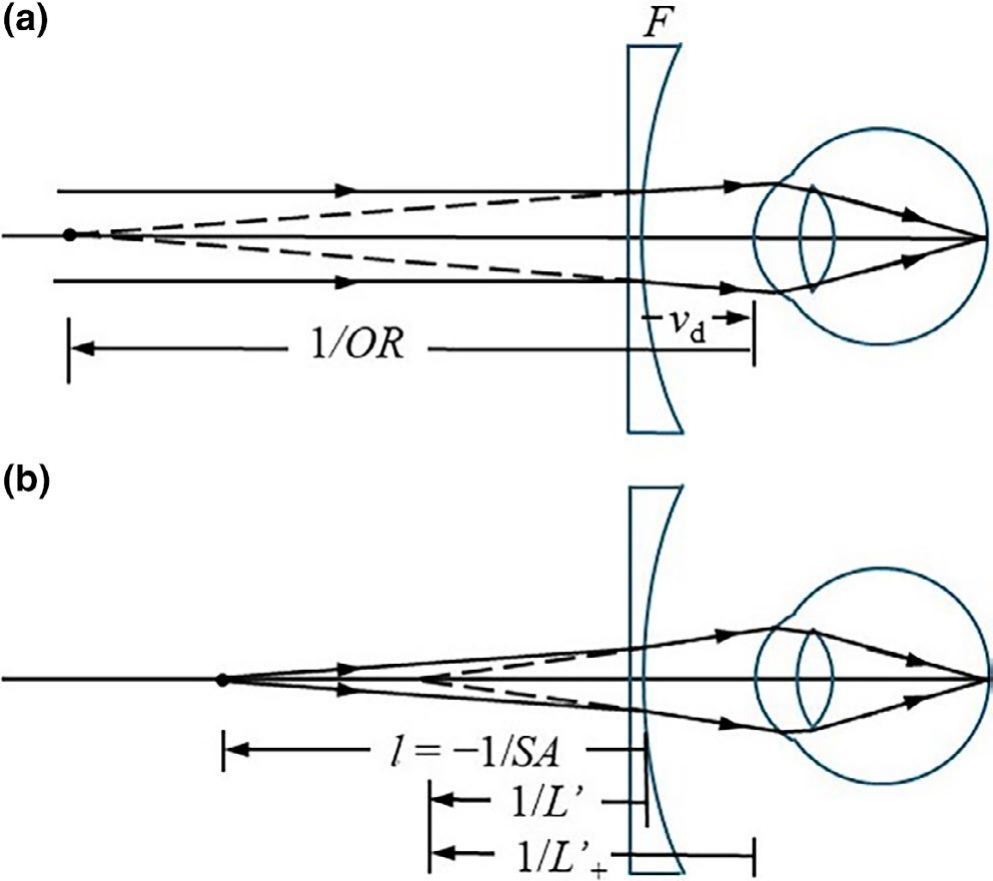
Accommodation parameters. (a) Spectacle refraction *F* and ocular refraction *OR*, (b) spectacle accommodation *SA*, image vergence leaving lens *L*' and image vergence at eye *L*'_+_. *l*, object distance; *V*_d_, vertex distance.

Another issue is how the accommodation (stimulus) is determined, as this can be referenced to the spectacle lens or the front of the eye. This is explained with the aid of Figure 1. Figure 1a shows an unaccommodated myopic eye viewing a distant object through a correcting lens of power *F* at a vertex distance *v*_d_ from the eye. This power is also referred to as the *spectacle refraction*. The refraction referenced to the front of the eye is the ocular refraction *OR*. It is given by

(7) $$\text{OR}=F/\left(1–v_{d}F\right)$$

Figure 1b shows the eye accommodated for a target (negative) distance *l* in front of the spectacle lens. The eye has a *spectacle accommodation SA* where

(8) SA=−1/l

After refraction at the lens, vergence is

(9) $$L^{\prime }=-\mathrm{SA}+F$$

Transfer to the eye gives a vergence

(10) $$\begin{array}{c} {L^{\prime }}_{+}=L^{\prime }/\left(1–v_{d}L^{\prime }\right)=\left(F-\mathrm{SA}\right)/\left[1–v_{d}\left(F-\mathrm{SA}\right)\right] \end{array}$$

The accommodation referenced to the eye is called *ocular accommodation*. It is given by

(11a) $$\begin{array}{c} \mathrm{OA}=\text{OR}-{L^{\prime }}_{+}=F/\left(1–v_{d}F\right)–\left(F-\mathrm{SA}\right)/\left[1–v_{d}\left(F-\mathrm{SA}\right)\right] \end{array}$$

For the emmetrope, for which *F* = 0, Equation (11a) reduces to

(11b) $$\mathrm{OA}=\mathrm{SA}/\left[1+v_{d}\mathrm{SA}\right)]$$

This is sufficient development for the purposes here. More detailed descriptions about the associations between accommodation terms are provided elsewhere.^21^, ^47^, ^48^

**FIGURE 2:**
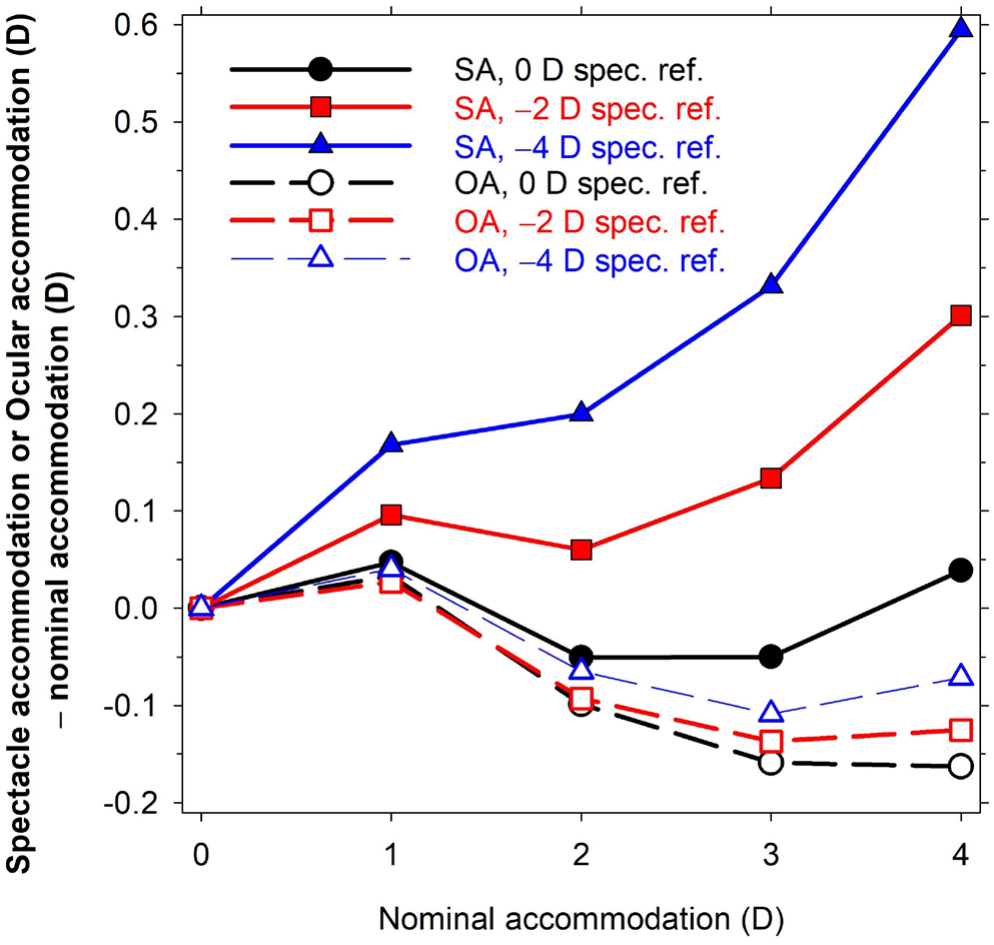
Difference between either spectacle (*SA*) or ocular accommodation (*OA*) and nominal accommodation, as a function of nominal accommodation for schematic eyes with different levels of spectacle refraction (0, −2 and −4 D). Vertex distance 12 mm.

The accommodation *A* according to the equations used in this work differs slightly from both the spectacle and ocular accommodation. It can be considered to be *nominal* accommodation as it was based on unaccommodated and accommodated emmetropic eyes. Although the accommodating changes to parameters of the myopic eyes are the same as for the corresponding emmetropic eyes, it cannot be expected that they would give exactly the same spectacle or ocular accommodation. This is shown in Figure 2, which plots (spectacle accommodation—nominal accommodation) and (ocular accommodation—nominal accommodation) as functions of nominal accommodation. The spectacle accommodation is up to 0.59 D more positive than the nominal accommodation (−4 D spectacle refraction), whilst the ocular accommodation is up to 0.16 D more negative than the nominal accommodation (0 D spectacle refraction). Even for the emmetropic eye, neither spectacle nor ocular accommodation is the same as the nominal equation because Equation (6) for the lens diameter does not provide perfect refraction solutions across the accommodation range. For the following results, ‘accommodation’ has been used rather than ‘nominal accommodation’ and ‘refraction’ or degree of myopia is used rather than ‘spectacle refraction’.

## RESULTS

### On‐axis spherical aberration

**FIGURE 3:**
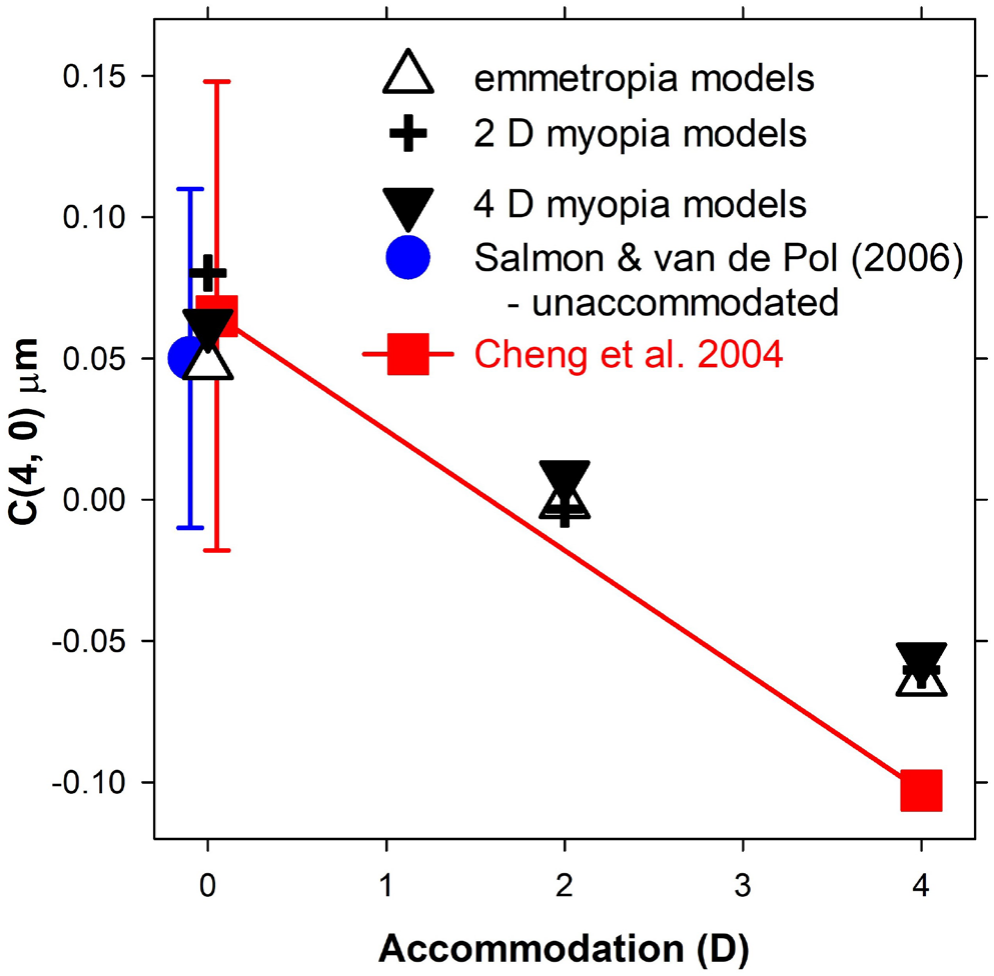
Spherical aberration coefficient *C*(4, 0) as a function of accommodation, for schematic eyes and results of Salmon and van de Pol^49^ and Cheng et al.^12^

Figure 3 shows the spherical aberration coefficient results. The spherical aberration coefficient for the 5 mm diameter pupil has values of +0.048, −0.001 and −0.064 μm for emmetropic unaccommodated eyes with 0, 2 and 4 D accommodation, respectively. For unaccommodated eyes, Salmon and van de Pol's compilation across 10 studies gave a value of 0.05 ± 0.06 μm, whilst Cheng et al. obtained 0.07 ± 0.08 μm,^12^, ^49^ so our unaccommodated result is similar to these findings. Cheng et al. determined that the coefficient changed as a function of the accommodation response at a mean rate of −0.042 μm/D, predicting −0.10 μm for 4 D accommodation. Based on the difference between the unaccommodated and accommodated rates, our rate of change was −0.028 μm/D or about 67% of that from Cheng et al. The thought was given to making the posterior surface more aspheric, but this detracts from the purpose of having similar peripheral refraction patterns for the unaccommodated and accommodated eyes.

The spherical aberration coefficients for 2 and 4 D myopic eyes are similar to those for the emmetropic eyes (Figure 3).

### Peripheral refraction

**FIGURE 4:**
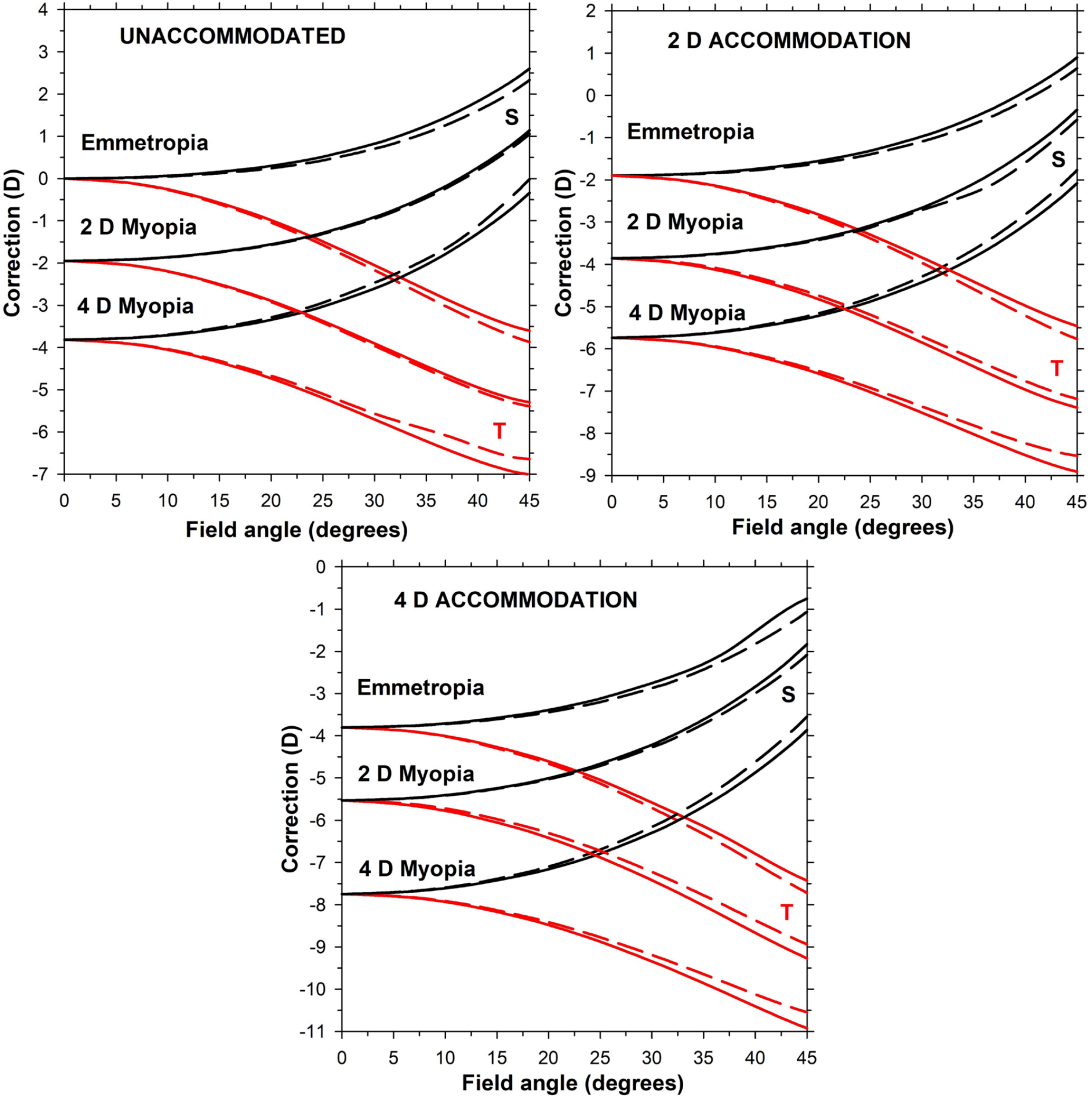
Sagittal and tangential peripheral corrections of the unaccommodated (top left), 2 D accommodated (top right) and 4 D accommodated forms (bottom) of the schematic eyes. Each part shows results for emmetropic, 2 D myopic and 4 D myopic eyes along the vertical field (continuous lines) and along the horizontal field (dashed lines). Note the shift of the correction scales between the different parts.

Figure 4 shows the peripheral refractions for eyes with spectacle refractions of 0, −2.0 and −4.0 D and accommodation levels of 0, 2 and 4 D referenced to the front of the eye. All eyes show positive sagittal correction and negative tangential correction shifts into the periphery. The myopic eyes have slightly more positive, or less negative, relative correction than their respective emmetropic eyes. The accommodated eyes show similar peripheral correction shifts as their respective unaccommodated eyes. Refractions are similar for corresponding vertical and horizontal fields.

## DISCUSSION/CONCLUSION

Schematic eyes attempt to represent ‘typical’ eyes and to be capable of predicting at least some aspects of real‐eye performance. In practice, of course, real eyes with nominally the same characteristics show a wide range in each of their specific individual biometric parameters and performance criteria. Clearly, customised models based on the measured parameters for the individual eye are likely to give more accurate predictions of aberrations and overall visual performance. Nevertheless, the 2006 refraction‐dependent, unaccommodated schematic eye^4^ stands up reasonably well when recent literature for the young adult eye is considered. Here we have extended it to develop a schematic model which gives reasonable predictions of spherical aberration and peripheral refraction over the accommodation range 0–4 D. It incorporates accommodation‐dependent changes to several lens‐related parameters: these are based on literature values.

We introduced changes in anterior chamber depth, lens thickness, vitreous chamber depth, lens surface radii of curvature and front surface asphericity. Next, whilst retaining a parabolic variation with relative distance from the lens centre and with unchanged edge and centre refractive indices, we manipulated the gradient index distribution together with the lens diameter to maintain focus on the retina for the emmetropic case. Finally, we changed the asphericity of the back surface so that spherical aberration and peripheral refraction approximately matched typical literature trends. The change in spherical aberration in the negative direction is less than that found in Cheng et al.^12^ Peripheral refraction does not vary much with the change from the unaccommodated to the accommodated state (in accordance with the literature). Although the results presented in Figures 3 and 4 are limited to eyes which are emmetropic, 2 D myopic and 4D myopic, with accommodation levels of 0, 2 and 4 D, it is straightforward to use the parametric equations of Table 1 to obtain results for other levels of myopia and accommodation within the range for which the schematic eye is valid.

As a comparison with the present studies, changes in the well‐known Navarro schematic eye^3^ were considered. The latter has parameters which vary as a function of the natural log of (*A* + 1), where *A* is the accommodation. When the expressions for the parameters are differentiated to find the rates of change with accommodation, this yields the factor (*A* + 1)^−1^, so that the rates of change vary with the accommodation level, as shown in the second‐to‐bottom line of Table 2. The bottom line of Table 2 shows the rates of change of the new accommodated eye (see Table 2), which do not vary with accommodation. Whilst the orders of magnitude for the two sets of predictions of changes are similar for modest levels of accommodation, there are considerable differences between the rates of change in the two schematic eyes. In part, this is because the Navarro eye uses a constant (or equivalent) refractive index for the lens for any level of accommodation rather than a changing index gradient.

As noted in the Introduction, one of the main motivations for developing the proposed eye was to evaluate retinal imagery when spectacles or other optical devices designed to inhibit the development of myopia are worn. Since such development usually occurs in childhood, a major weakness of the model is that it refers to young adults, rather than to children. In the longer term, therefore, there is a need for a childhood‐based schematic model which includes parameter variations with age as well as the variables of refraction and accommodation which form part of the model presented here. An initial step in this direction was made by Li et al.,^50^ who developed refraction‐dependent schematic eyes for 7‐year and 14‐ year‐old Chinese children. Refraction‐related parameters were axial length and component lengths except for corneal thickness, as well as corneal and lens powers. Gender‐related parameters were axial length, component lengths and anterior corneal radius of curvature. Age‐related parameters were axial and component lengths.

Whilst in principle extension to include higher levels of myopia and hyperopic eyes is possible, available biometric data are sparse, and the validity of assuming linear relationships between parameters and spectacle refraction over a wider range has not yet been explored.

In conclusion, the wide‐angle model presented here extends the range of schematic eyes to include both refraction and accommodation as variable input parameters. It may be useful in predicting aspects of retinal image quality.

## AUTHOR CONTRIBUTIONS

**David A. Atchison:** Conceptualization (equal); formal analysis (lead); investigation (lead); methodology (lead); resources (lead); writing – original draft (equal). **W. Neil Charman:** Conceptualization (equal); writing – original draft (equal).

## FUNDING INFORMATION

None.

## CONFLICT OF INTEREST STATEMENT

All authors of this article declare no conflict of interest.
